# Perceived Usefulness of a Microfinance Intervention on Health Awareness and Practices in Nepal

**DOI:** 10.3389/fpubh.2015.00289

**Published:** 2016-01-14

**Authors:** Bharat Ram Dhungana, Jitendra Kumar Singh, Dilaram Acharya, Salila Gautam, Pravin Paudyal

**Affiliations:** ^1^School of Business, Pokhara University, Pokhara, Nepal; ^2^Department of Community Medicine, Janaki Medical College, Tribhuvan University, Janakpur, Nepal; ^3^Department of Community Medicine, Devdaha Medical College and Research Institute, Kathmandu University, Devdaha, Nepal; ^4^Department of Public Health, Sanjeevani College of Medical Sciences, Purbanchal University, Butwal, Nepal; ^5^Clinical and Research Department, Everest International Clinic and Research Centre, Kathmandu, Nepal

**Keywords:** ethnicity, health awareness, health practices, microfinance, Nepal

## Abstract

**Background:**

Economic constraints may lead to poor health among people from the developing world. Microfinance has proven to be useful in improving health outcomes elsewhere, but it still remains a neglected issue in Nepal. This study aims to assess perceived usefulness of the microfinance on health awareness and practices among different ethnic groups of Nepal.

**Method:**

A community-based cross-sectional study was conducted in four districts of western Nepal. A total of 500 microfinance clients representing different ethnic groups (upper caste, aadibasi and janajati, and dalit) were selected by using systemic random sampling. Health awareness and practices among different ethnic groups were compared by logistic regression after adjustment for age, level of education, sex of household heads, occupation, and place of residence. Since participants were asked about their health awareness and practices, before and after microfinance intervention, during a single interview, there was a strong possibility of recall bias with respect to their preintervention awareness and other measures.

**Results:**

Microfinance intervention positively influenced self-reported health awareness and practices among different ethnic groups in Nepal, which was highest among the upper caste group (77–92%, rate ratios around 1.7–2.6), followed by the aadibasi/janajati (60–76%, rate ratios around 1.4–1.8) and dalit group (33–52%, reference group). Self-reported awareness about environment and sanitation, family planning methods, and available health services at local level improved from 11.2 to 69.2, 9.2 to 65.0, and 3.8 to 59.8%, respectively, among the clients after microfinance intervention (*p* < 0.001). Similarly, the practices of eating nutritious food/balanced diet, drinking safe water, using toilet, immunizing the children, and regularly visiting the healthcare facility were improved from 4.2 to 63.8, 12.6 to 66.8, 15.2 to 70.4, 15.8 to 73.8, and 3.6 to 61.4%, respectively (*p* < 0.001).

**Conclusion:**

Despite lack of control group and potential recall bias, this study reports positive effect of microfinance on self-reported health awareness and practices among different ethnic groups of Nepal. This finding supports further implementation and evaluation of equity-based microfinance to improve health and economic conditions of Nepalese people.

## Introduction

Every year, 25 million households (more than 100 million people) are pushed into poverty due to recurrent illnesses and expensive healthcare. Access to the good health services and health protection are key components for fighting against poverty (1, 2). Microfinance refers to a provision of financial services such as loans, savings, and business trainings for the poor (3). In Asian context, formal microfinance institutions (MIFs) are generally regulated under the banking legislation and are supervised by central banks (4). As health and poverty are interrelated to each other (5), improving health of the poor necessitates poverty reduction and an increased access to healthcare.

Microfinance organizations can implement health programs that increase knowledge, change health-related behaviors, and improve access to health services (6). Microfinance has been shown to have a positive impact on many health issues, such as malaria and tuberculosis control (7, 8), maternal (9), and child health (10). In sub-Saharan Africa, microfinance improved both the financial and various non-financial aspects such as health, nutrition, food security, education, child labor, women’s empowerment, housing, job creation, and social cohesion (11). Microfinance was also found to similarly improve population health and value for money for such programs in South Asia (12).

Nepal is a landlocked country with per capita income of just US$ 703 in 2013, and 23.8% of its population still live below the poverty line (13). Nepalese banks and financial institutions have been classified as commercial banks (class A), development banks (class B), finance companies (class C), and microfinance development banks (class D) (14, 15). Savings and Credit Cooperatives (SCCs), Small Farmers’ Cooperatives Limited (SFCLs), Financial Intermediary NGOs (FI-NGOs), and Microfinance Development Banks are the four major active types of MIs in Nepal (16). Nepal’s microfinance sector has expanded significantly to provide services to its clients through the outreach services (14, 15, 17). Despite the hostile topography of Nepal, microfinance aims to alleviate poverty through rural development. It has focused on very poor and addresses the challenges of sustainability and outreach (16, 18). A previous Nepalese study on the impact of microfinance services for women’s socioeconomic empowerment found a positive effect on women’s participation in community work, healthcare, children’s education, and their speaking and decision-making abilities (19). However, to the best of our knowledge, no research about the effect of membership-based microfinance intervention on health awareness and practices in Nepalese population has ever been performed and published. Therefore, the current research aimed to assess the perceived usefulness of the microfinance on health awareness and practices among the different ethnic groups of Nepal.

## Materials and Methods

### Study Setting

The survey included 500 microfinance clients from four districts (Nawalparasi, Kaski, Parbat, and Baglung) of western development region, which consist of 300 government microfinance institutions (GMFIs) clients and 200 private MFIs (PMFIs) clients who were involved regularly in microfinance program for last 5 years. This research includes the “D class” MFIs working in western development region of Nepal (14, 15). Data were collected between September 2, 2013 and February 3, 2014.

### Study Design and Sampling

**Figure 1:**
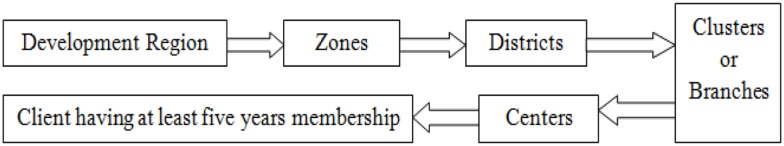
**Clients having at least 5 years membership**.

A cross-sectional survey was used in this study. The multistage cluster sampling method was applied by covering a wide geographical area to ensure the representation from each zone of the western development region of Nepal in sample as shown in Figure 1 and Table 1.

**Table 1:** **Distribution of population and sample size**.

Zone	District	Types of microfinance institutions^a^	Branch^b^	Number of centers	Sampled center	Total number clients of the sampled center (having at least 5 years membership in MFIs)	Sample size	
							Clients	%
Lumbini	Nawalparasi	PAGBB	Kawasoti	34	5	135	32	23.7
		PAGBB	Beldiha	22	4	112	23	20.5
		PAGBB	Chormara	26	5	134	35	26.1
		NUBL	Daldale	96	10	138	40	29.0
		DLBB	Parasi	55	9	128	30	23.4
Gandaki	Kaski	PAGBB	Lekhnath	56	7	146	42	28.8
		PAGBB	HB	60	4	155	38	24.5
		NUBL	SC	106	12	175	60	34.3
		DLBB	IC	119	15	145	37	25.5
		DLBB	Bagar	72	8	124	33	26.6
Dhawalagiri	Parbat	PAGBB	Parbat	25	9	182	50	27.5
	Baglung	PAGBB	Baglung	27	8	241	80	33.2
3	4	3	12	698	96	1,815	500	27.5

*^a^PAGBB, Paschimanchal Grameen Bikas Bank (government-owned MFI); NUBL, Nirdhan; Utthan Bank Limited (private-owned MFI); DLBB, Deprosc Laghubitta Bikas Bank (private-owned MFI)*.

*^b^HB, Hemja-Bagar; SC, Shrijana Chok, and IC, Indra Chok*.

The survey included the sample size of 500 microfinance clients from four districts (Nawalparasi, Kaski, Parbat, and Baglung) of western development region of Nepal, which was estimated using formula for estimating the optimal sample size (20).

$$n=\frac{Z^{2}\times p\times q\times N}{e^{2}\left(N-1\right)+Z^{2}\times p\times q}$$

where *n* = optimal sample size, *N* = size of population, *Z* = value of standard variant at 5% level if confidence interval = 1.96, *p* = variability (variability has been assumed 0.5), *q* = 1 − *p*, and *e* = level of precision or acceptable sampling error tolerated (assumed 5.0%).

Then,

$$\begin{array}{c} n=\frac{Z^{2}\times p\times q\times N}{e^{2}\left(N-1\right)+Z^{2}\times p\times q}=\frac{{\left(1.96\right)}^{2}\times 0.5\times 0.5\times 1,815}{{\left(0.05\right)}^{2}\left(1,815-1\right)+{\left(1.96\right)}^{2}\times 0.5\times 0.5}=317 \end{array}$$

The clients, who are involved in different clusters of MIFs for five or more years, are the total population (*N*) for the clients’ survey. The sample size before adjustment of design effect was 317. The sample size was adjusted to account for intracluster correlation. The adjusted *n* = *n* × design effect. Here, the design effect 1.5 was taken as recommended for social rating. Thus, adjusted sample size after incorporating design effect was 476. Also, by allowing the 5% potential non-response, we inflated the final sample size to 500. All the participants were users of microfinance services with membership of MIF. Out of 565 such membership-based microfinance clients visited, 65 did not consent for an interview with 88.5% of response rate.

### The Intervention

The interventions were related to financial and non-financial aspects of microfinance services taken by respondents exactly 5 years prior to the data collection. The financial services were microcredit, micro-savings, microenterprises, or business creation. The non-financial services were the women empowerment and awareness creation in terms of health awareness and practices, education, and other social dimension provided during monthly meetings of MIFs.

### Data Collection

The research was based on primary data. The primary data were collected by structured questionnaire during face-to-face interviews. MFIs had a list of clients who had been getting microfinance services for at least 5 years. Based on these institutional information, research assistants visited clients and conducted interviews. Participants were interviewed about their health awareness and practices before and after microfinance intervention during the same interview.

The English version of the questionnaire was prepared, checked, pretested in the neighboring district and necessary modifications were made as required. It was also translated to Nepali and back translated to English to ensure that the meanings of the questions were not altered. The final approval of the questionnaire was obtained during submission of PhD proposal in the Department of Commerce, Banaras Hindu University, India. The questionnaire consisted of two parts: (i) respondent’s general characteristics and (ii) health awareness and practices of respondents before and after a microfinance intervention. Seven research assistants, who were trained on questionnaires, conducted the interview and they were constantly supervised.

### Ethics

The research proposal was approved by the Institutional Review Board, Banaras Hindu University, India. Respondents provided consent before the interview. Personal identifiers were removed before data analysis.

### Definition of Variables

The outcome variables of the study were health awareness and health practices. Health awareness included three variables: awareness of environment and sanitation, family planning methods, and available health services at their local levels. Health practices included use of nutritious foods/balanced diet, use of safe drinking water, use of toilet, use of traditional healers when they get sick, immunization of children, and visits to health facility for heath checkup. All these variables had Yes or No responses.

Five confounding variables were defined as follows. Respondents’ ages were categorized into four groups (20–29, 30–39, 40–49, and 50 or above years). Education status was recorded as illiterate, literate only, primary, secondary, and above secondary levels. Respondents’ household heads were male or female. Occupation was coded as livestock, agriculture, business, and others (labor, daily wages). Place of residence was categorized as rural and urban.

Ethnicity was based on the caste system in Nepal and was divided into three major groups based on available literature and similarities between the caste/ethnic groups: (i) advantaged upper caste, (ii) disadvantaged aadibasi/janajati (J&A), and (iii) disadvantaged dalit. The advantaged group included Brahmin, Chhetri, Thakuri, and Sanyasi. The disadvantaged J&A included indigenous groups of Nepal. Disadvantaged dalit included the castes that were considered lower in the caste-based stratification within the Nepalese society (21).

### Statistical Analysis

The data were analyzed with the help of descriptive and inferential statistics mainly chi-square test and logistic regression methods to analyze the health awareness and practices of the clients by Nepalese ethnicities. Odds ratios were converted into rate ratios with adjustment of confounding variables (22). A *p* value ≤0.05 was considered statistically significant. Data were analyzed using Statistical Package for Social Sciences (SPSS version 17 for windows).

## Results

Table 2 presents the sociodemographic characteristics of the respondents. Majority of the dalit (73.7%), aadibasi/janajati (73%), and upper caste (72.3%) were from 30 to 49 years of age. Two-fifth of the dalit (40.1%) were illiterate compared to only (22.8%) aadibasi/janajati and (10.1%) upper caste. Majority of the household heads were males among all ethnic groups. Slightly more than half of dalit (52.6%) had occupation of livestock and agriculture compared to aadibasi/janajati (29.7%) and upper caste (30.4%). Majority of dalit (59.1%) were from rural area compared to aadibasi/janajati (46.0%) and upper caste (50.7%).

**Table 2:** **Sociodemographic characteristics of the respondents**.

Characteristics	Ethnicity			Total
	Dalit	Aadibasi/janajati	Upper caste	
**Age**				
20–29	19 (13.9)	26 (12.1)	23 (15.5)	68 (13.6)
30–39	56 (40.9)	85 (39.5)	63 (42.6)	204 (40.8)
40–49	45 (32.8)	72 (33.5)	44 (29.7)	161 (32.2)
≥50 years	17 (12.4)	32 (14.9)	18 (12.2)	67 (13.4)
**Education**				
Illiterate	55 (40.1)	49 (22.8)	15 (10.1)	119 (23.8)
Literate only	15 (10.9)	34 (15.8)	19 (12.8)	68 (13.6)
Primary	52 (38.0)	73 (34.0)	48 (32.4)	173 (34.6)
Secondary	15 (10.9)	49 (22.8)	57 (38.5)	121 (24.2)
Above secondary	0 (0.0)	10 (4.7)	9 (6.1)	19 (3.8)
**Household head**				
Male	125 (91.2)	189 (87.9)	131 (88.5)	445 (89.0)
Female	12 (8.8)	26 (12.1)	17 (11.5)	55 (11.0)
**Occupation**				
Livestock	52 (38.0)	39 (18.1)	24 (16.2)	115 (23.0)
Agriculture	20 (14.6)	25 (11.6)	21 (14.2)	66 (13.2)
Business	54 (39.4)	130 (60.5)	95 (64.2)	279 (55.8)
Others	11 (8.0)	21 (9.8)	8 (5.4)	40 (8.0)
**Residence**				
Rural	81 (59.1)	99 (46.0)	75 (50.7)	255 (51.0)
Urban	56 (40.9)	116 (54.0)	73 (49.3)	245 (49.0)
Total	137 (100)	215 (100)	148 (100)	500 (100.0)

*Source: Field Survey, 2014*.

Self-reported health awareness and practices of the respondents after 5 years of membership-based microfinance intervention is shown in Table 3. Awareness about environment and sanitation, family planning methods, and available health services at local level seemed to increase from 11.2 to 69.2%, 9.2 to 65.0%, and 3.8 to 59.8%, respectively among clients after the microfinance intervention (*p* < 0.001). Similarly, the use of nutritious food/balanced diet, safe drinking water, toilet, immunization of children, and regular visits to health facility were found to increase from 4.2 to 63.8, 12.6 to 66.8, 15.2 to 70.4, 15.8 to 73.8, and 3.6 to 61.4%, respectively (*p* < 0.001). However, use of traditional healers seemed to decrease from 15.6 to 1.4% (*p* < 0.001). Among the three ethnic groups, the highest rates of health awareness and practices were observed among the upper caste group (77–92%), followed by the aadibasi/janajati group (60–76%) and the dalit group (33–52%).These results altogether indicate toward a positive effect of microfinance intervention in the study population.

**Table 3:** **Self-reported health awareness and practices of respondents (*N* = 500) before and after microfinance intervention^a^**.

Variable/ethnicity	Response	Intervention		*p*-Value
		Before *n* (rate, %)	After *n* (rate, %)	
Awareness of environment and sanitation	Yes	56 (11.2)	346 (69.2)	<0.001
Dalit		8 (5.8)	61 (44.5)	<0.001
Aadibasi/janajati		22 (10.2)	149 (69.3)	<0.001
Upper caste		26 (17.6)	136 (91.9)	<0.001
Awareness of family planning methods	Yes	46 (9.2)	325 (65.0)	<0.001
Dalit		4 (2.9)	53 (38.7)	<0.001
Aadibasi/janajati		20 (9.3)	140 (65.1)	<0.001
Upper caste		22 (14.9)	132 (89.2)	<0.001
Awareness of available health services at local level	Yes	19 (3.8)	299 (59.8)	<0.001
Dalit		2 (1.5)	52 (38.0)	<0.001
Aadibasi/janajati		9 (4.2)	133 (61.9)	<0.001
Upper caste		8 (5.4)	114 (77.0)	<0.001
Use of nutritious food/balanced diet	Yes	21 (4.2)	319 (63.8)	<0.001
Dalit		1 (0.7)	54 (39.4)	<0.001
Aadibasi/janajati		10 (4.6)	139 (64.7)	<0.001
Upper caste		10 (6.7)	126 (85.1)	<0.001
Use of safe drinking water	Yes	63 (12.6)	334 (66.8)	<0.001
Dalit		10 (7.3)	56 (40.9)	<0.001
Aadibasi/janajati		26 (12.1)	148 (68.8)	<0.001
Upper caste		27 (18.2)	130 (87.8)	<0.001
Use of toilet	Yes	76 (15.2)	352 (70.4)	<0.001
Dalit		10 (7.3)	60 (43.8)	<0.001
Aadibasi/janajati		32 (14.9)	156 (72.6)	<0.001
Upper caste		34 (23.0)	136 (91.9)	<0.001
Use of traditional healers	Yes	78 (15.6)	7 (1.4)	<0.001
Dalit		44 (32.1)	3 (2.2)	<0.001
Aadibasi/janajati		27 (12.6)	3 (1.4)	<0.001
Upper caste		7 (4.7)	1 (0.7)	0.07
Immunization of children	Yes	79 (15.8)	369 (73.8)	<0.001
Dalit		12 (8.8)	70 (51.1)	<0.001
Aadibasi/janajati		32 (14.9)	163 (75.8)	<0.001
Upper caste		35 (23.7)	136 (91.9)	<0.001
Regular visit to health facility for health checkup	Yes	18 (3.6)	307 (61.4)	<0.001
Dalit		1 (0.7)	45 (32.8)	<0.001
Aadibasi/janajati		7 (3.2)	131 (60.9)	<0.001
Upper caste		10 (6.7)	131 (88.5)	<0.001

*^a^Health awareness and practices before and after microfinance intervention were taken from a single interview for all the respondents who were clients of microfinance up to 5 years after the start of the intervention*.

Multivariable analysis of self-reported health awareness and practices in relation to ethnicity of the respondents after microfinance intervention has been demonstrated in Table 4. Compared with the dalit group, the rate ratios related to health awareness and practices for the aadibasi/janajati group ranges from 1.4 to 1.8 and for the upper caste group 1.7 to 2.6. Rate ratio for the use of traditional healers among the aadibasi/janajati group was around 0.6, and for the upper caste groups, the rate ratios was around 0.3 as compared to dalit.

**Table 4:** **Multivariable analysis of self-reported health awareness and practices in relation to ethnicity of the respondents after microfinance intervention**.

Dependent variables	Ethnicity	Rate ratio (RR)	95% CI
Awareness of environment and sanitation	Dalit	1.0	1.00
	Aadibasi/janajati	1.5	1.1–2.1
	Upper caste	2.0	1.5–2.8
Awareness of family planning methods	Dalit	1.0	1.00
	Aadibasi/janajati	1.6	1.2–2.3
	Upper caste	2.3	1.6–3.2
Awareness of available health services at local level	Dalit	1.0	1.00
	Aadibasi/janajati	1.6	1.1–2.2
	Upper caste	2.0	1.4–2.8
Use of nutritious food/balanced diet	Dalit	1.0	1.00
	Aadibasi/janajati	1.6	1.1–2.2
	Upper caste	2.1	1.5–3.0
Use of safe drinking water	Dalit	1.0	1.00
	Aadibasi/janajati	1.6	1.2–2.3
	Upper caste	2.1	1.5–2.9
Use of toilet	Dalit	1.0	1.00
	Aadibasi/janajati	1.6	1.2–2.2
	Upper caste	2.0	1.5–2.8
Use of traditional healers	Dalit	1.0	1.00
	Aadibasi/janajati	0.6	0.08–4.7
	Upper caste	0.3	0.005–3.8
Immunization of children	Dalit	1.0	1.00
	Aadibasi/janajati	1.4	1.1–1.9
	Upper caste	1.7	1.3–2.4
Regular visit to health facility for health checkup	Dalit	1.0	1.00
	Aadibasi/janajati	1.8	1.3–2.6
	Upper caste	2.6	1.9–3.8

## Discussion

Previously, health benefits, in addition to financial and other non-financial benefits of microfinance, have been reported in sub-Saharan Africa and South Asia (11, 12). Our current study indicates that microfinance intervention might improve the components of awareness and practices related to health except for the use of traditional healers. However, this study could not completely rule out the possibility of recall bias with respect to their preintervention awareness and other measures. Similarly, the positive impact of microfinance on knowledge of maternal and child health has been demonstrated in the studies from Bangladesh and Indonesia (9, 10). This could be due to the increased access to health information to the clients as well as their improved economic status after microfinance intervention. Many microfinance organizations have implemented health programs to increase knowledge about health-related behaviors and improve access to health services (6). The study suggests that the incorporation of microfinance strategy to health strategy could possibly be effective in developing countries like Nepal.

Interestingly, use of traditional healers to manage health problems in this study was significantly lowered after microfinance interventions. Moreover, the highest level of health awareness and practices were observed among microfinance clients of upper caste group, followed by aadibasi/janajati group and the dalit group. This could be due to the increased uptake of health information, higher education level, and better communication and economic benefits among upper caste. Based on these observations, it is presumed that an equity-based microfinance services with more emphasis to the lower caste groups would be more effective in Nepal.

Our study indicates that awareness of environmental health and sanitation, different family planning methods, and availability of health services at local level were improved after microfinance intervention among upper caste group more commonly than others. The findings of current study are in line with other studies from India and Bangladesh (23, 24). Improved health awareness and an increased access to formal health care through microcredit have also been shown in the study from Bangladesh (25).

This study indicates a significant improvement in the use of nutritious diet, safe drinking water, toilet, health checkup at local health facility, immunization of their children after the microfinance membership-based intervention. Some previous literatures reported that access to financial services improved health conditions, such as nutrition, higher immunization rates, and child health (10, 26). In Ghana, the use of maternal and child health interventions, such as skilled care at birth, intranatal care, use of modern contraceptives, and malaria control, were more common in the rich population as compared to the poor (8). A Dominican Republic study also reported an improvement in water and sanitation as well as maternal and child health indicators through microcredit and health promotion programs. Moreover, significant improvement in 10 out of 11 such indicators was reported by the same study (27). Another Bangladeshi study also confirmed that household’s relative poverty status, as reflected by wealth quintiles, was a major determinant in health-seeking behavior. Mothers who were of the highest wealth quintile were significantly more likely to use modern trained providers of antenatal care, birth attendance, postnatal care, and child health care than the mothers in the lowest wealth category (28).

Our results suggest that microfinance has been useful to improve health awareness and practices. To the best of our knowledge, no such previous study has been conducted on effect of microfinance on health awareness and practices in Nepal. Nevertheless, despite our best effort, this study also has some limitations. First, due to the lack of a control group, the methods do not allow to assess whether the improvements in health awareness and practices are indeed due to the intervention. This also holds true for the differences between the three subpopulations. In particular, it is not clear how the intervention has affected equity. Although the study explored an important area and the current observations are encouraging, better-controlled experimental designs would provide stronger evidence for the effectiveness of microfinance. Second, there was potential for a recall bias in data collection, particularly for the data related to outcome measures before the intervention as one set of interviews was conducted after the intervention had taken place.

## Conclusion

This study found positive effects of microfinance on health awareness and practices in different ethnic groups of Nepal. The positive health awareness and practice were more commonly observed among upper caste group. Therefore, the study indicates toward a need for an equity-based microfinance intervention for improving the health and economic status of the Nepalese people. Additional follow-up interventional studies are recommended in this regard.

## Author Contributions

JS and BD participated in the design of the study. DA and JS performed statistical analysis. DA and JS wrote manuscript with significant contribution of BD and SG. BD, SG, and PP contributed in the analysis, interpretation of the results, and literature review. All of the authors contributed in revision and agreed on the final manuscript.

## Conflict of Interest Statement

The authors declare that the research was conducted in the absence of any commercial or financial relationships that could be construed as a potential conflict of interest.
